# The efficacy of Narrative Exposure Therapy for Children (KIDNET) as a treatment for traumatized young refugees versus treatment as usual: study protocol for a multi-center randomized controlled trial (YOURTREAT)

**DOI:** 10.1186/s13063-020-4127-4

**Published:** 2020-02-14

**Authors:** Sarah Wilker, Claudia Catani, Jasmin Wittmann, Melissa Preusse, Telja Schmidt, Theodor May, Verena Ertl, Bettina Doering, Rita Rosner, Areej Zindler, Frank Neuner

**Affiliations:** 1 Clinical Psychology and Psychotherapy, Bielefeld University, Universitätsstraße 25, 33615 Bielefeld, Germany; 2 Independent Biostatistician, Johann-Strauß-Str. 11a, 33647 Bielefeld, Germany; 3 Clinical Psychology and Biopsychology, Catholic University Eichstätt-Ingolstadt, 85071 Eichstätt, Germany; 4 Medical Center Hamburg-Eppendorf, Outpatient Clinic for Refugee Children and Adolescents, Martinistraße 52, 20246 Hamburg, Germany

**Keywords:** Post-traumatic stress disorder, Children, Adolescents, Refugees, Narrative Exposure Therapy for Children (KIDNET), Randomized controlled trial

## Abstract

**Background:**

Germany hosts a large number of refugees from war-affected countries. The integration of refugees, in particular young refugees from the Middle East, is one of the major current social challenges in Germany. Mental disorders, first of all post-traumatic stress disorder (PTSD) that results from war experiences, are common among young refugees and interfere with quality of life as well as functional integration. Evidence regarding effective treatment options for this population is scarce. In this trial, we aim to evaluate the pragmatic, short-term psychotherapy Narrative Exposure Therapy for Children (KIDNET) for the treatment of young refugees in Germany.

**Methods:**

In a rater-blinded, multi-center, randomized-controlled trial, KIDNET is compared to treatment as usual (TAU) within the general health care system. A total number of 80 young refugees who fulfill the diagnostic criteria of PTSD will be randomized to either KIDNET or TAU. Diagnostic interviews will take place at baseline before treatment as well as 6 and 12 months thereafter. They will assess exposure to traumatic events, PTSD and comorbid symptoms, as well as parameters of integration.

**Discussion:**

The results of this study should provide evidence regarding effective treatment options for young refugees in Germany, a population that has been understudied and received only limited access to mental health care so far. Next to the effects of treatment on mental health outcomes, integration parameters will be investigated. Therefore, this study should provide broad insights into treatment options for young refugees and their potential implications on successful integration.

**Trial registration:**

German Clinical Trials Register (Deutsches Register Klinischer Studien; DRKS), ID: DRKS00017222. Registered on 15 May 2019.

## Background

Germany is host to a large number of refugees from various war-torn countries. In the home countries as well as on their dangerous journey to Germany, refugees have experienced numerous traumatic experiences. Since the likelihood to develop mental health disorders increases with accumulating traumatic events in a dose-dependent manner [1, 2], a high prevalence of mental ill-health can be expected in this population, in particular high rates of post-traumatic stress disorder (PTSD) and comorbid disorders. So far, evidence regarding the prevalence rate of mental health disorders of refugees in Germany is limited. However, an initial study indicates that approximately one half of refugees living in a German refugee accommodation were screened positive for mental health problems [3]. In a representative sample of Syrian refugee children from a German reception camp, PTSD was diagnosed in 33% of children aged 7–14 years [4].

Although the majority of trauma survivors recover without treatment over time, war-related PTSD seems to be highly persistent [5]. PTSD is characterized by symptoms like concentration difficulties, loss of interest in social activities, insomnia and irritability that involve a high impairment in everyday functioning [6]. Furthermore, higher PTSD symptom severity is associated with an increased risk of poor physical health [7]. In addition, PTSD symptoms in children and adolescents are correlated with neuropsychological dysfunctions in a range of cognitive tasks [8, 9] as well as with reduced school performance [10]. PTSD symptoms but not trauma exposure are related to reduced intellectual capabilities [9] in particular in verbal skills [11]. A study with war-affected children in Sri Lanka found that children with PTSD presented with lower school grades in language-related subjects [12]. In a treatment-seeking sample of refugees in Switzerland, the severity of PTSD symptoms was associated with social integration difficulties [13]. Taken together, it is likely that next to the individual suffering, PTSD symptoms interfere with integration into the host country, as well as academic achievement. Consistent with this assumption, a study in Sweden has shown that Iraqi refugees with PTSD had a significantly delayed performance in language schools [14]. In the face of the expectedly high prevalence rate of PTSD in the current refugee population and the probable interference of PTSD with integration, the development of effective, evidence-based and pragmatic treatments for PTSD, especially for these populations, should be a public health priority.

Cumulating evidence from clinical studies indicates that the different variants of the so-called trauma-focused psychotherapies are the treatments of first choice for PTSD in children [15]. Trauma-focused treatments have in common that they target traumatic memories through exposure or cognitive interventions. A recent independent evaluation indicated that, on the basis of the rationale as well as the available efficacy, Narrative Exposure Therapy (NET) is probably advantageous for the treatment of traumatized refugees in comparison to other trauma-focused approaches [16]. However, current evidence is limited with regard to the treatment of war-affected *children* with PTSD. The available knowledge relies on case-series and cohort studies with only a few exceptional randomized trials. A systematic literature research in MEDLINE identified 12 published randomized controlled trials (RCTs) with war-affected children (see Table 1). Out of these, five studies reported the effectiveness of school-based cognitive behavior therapy (CBT)-like interventions that did not screen for or exclude children with high values of PTSD symptoms (four of these did not find a significant effect on PTSD symptoms), one screened youths for distress and did also not find an effect on PTSD, two applied trauma-focused-CBT in groups and one applied individual trauma-focused-CBT and found a significant effect on PTSD. Three trials studied the effectiveness of Narrative Exposure Therapy for Children (KIDNET), also with a significant effect on PTSD symptoms. Only a single RCT studied PTSD treatment (KIDNET) in refugee children [26].

**Table 1:** Results of a systematic literature search in MEDLINE on 16 February 2018

Study	Population	Treatment	Control	Findings
Dawson et al. 2017 [17]	Indonesian children, 7–14 years, screened for PTSD	Individual tf-CBT	Problem-solving	Significant reduction of PTSD in both groups
Ooi et al. 2016 [18]	War-affected child migrants in Australia, 10–17 years, screened for PTSD, only individuals with mild to moderate PTSD symptoms included	TRT (CBT based, in group)	Waiting list	No effect on PTSD symptoms
Betancourt et al. 2014 [19]	Sierra Leone youth, 14–24 years, screened for distress, not for PTSD	CBT based, no trauma focus	Waiting list	No effect on PTSD symptoms
Tol et al. 2014 [20]	Burundi children, 8–17 years, no specific screening	School-based intervention, no trauma focus	Waiting list	No effect on PTSD symptoms
McMullen et al. 2013 [21]	Boys in Congo, 13–17 years, screened for war exposure	tf-CBT in group	Waiting list	Significant effect on PTSD symptoms
O’Callaghan et al., 2013 [22]	Girls in Congo, 12–17 years, screened for sexual violence	tf-CBT in group	Waiting list	Significant effect on PTSD symptoms
Tol et al., 2012 [23]	Children in Sri Lanka, 9–12 years, no specific screening	School-based intervention, no trauma focus	Waiting list	No effect on PTSD symptoms
Qouta et al., 2012 [24]	Children in Palestine, 10–13 years, no specific screening	School-based intervention, no trauma focus	Waiting list	Only overall effect on PTSD symptoms
Ertl et al., 2011 [25]	Former child soldiers in Uganda, 12–25 years, with PTSD	NET	Waiting list, academic counseling	Significant reduction of PTSD symptoms, superiority of NET
Ruf et al. 2010 [26]	Refugee children in Germany, 7–16 years, with PTSD	NET	Waiting list	Significant reduction of PTSD symptoms, superiority of NET
Catani et al. 2009 [27]	Sri Lankan children after war and tsunami, 8–14 years, with PTSD	NET	Meditation	Significant reduction of PTSD in both groups
Layne et al. 2008 [28]	Bosnian children and youth, 13–19 years, with major PTSD symptoms	Trauma-focused school-based intervention	Psychoeducation and skills	Reduction of PTSD in both groups, Superiority of trauma-focus

We used the keywords (“warfare”[MeSH Terms] OR “warfare”[All Fields] OR “war”[All Fields]) AND (“child”[MeSH Terms] OR “child”[All Fields] OR “children”[All Fields]) AND (“stress disorders, post-traumatic”[MeSH Terms] OR (“stress”[All Fields] AND “disorders”[All Fields] AND “post-traumatic”[All Fields]) OR “post-traumatic stress disorders”[All Fields] OR “ptsd”[All Fields]) AND (“random allocation”[MeSH Terms] OR (“random”[All Fields] AND “allocation”[All Fields]) OR “random allocation”[All Fields] OR “randomized”[All Fields])

Abbreviations: *CBT* cognitive behavior therapy, *NET* Narrative Exposure Therapy, *PTSD* post-traumatic stress disorder, *tf-CBT* trauma-focused cognitive behavior therapy, *TRT* Teaching Recovery Techniques

A recent independent review [29] confirmed that, consistent with the literature from war-affected adults [30], KIDNET is one of the most promising and best-studied treatment approaches for war-affected children with PTSD and has generally caused a clinically significant improvement in treated children.

In the three available KIDNET trials that included children and adolescents, KIDNET has been compared to meditation for school children in Sri Lanka [27], academic counseling and a waiting-list control condition in former child-soldiers in Uganda [25], as well as a waiting-list control group in asylum seekers in Germany [26]. Taken together, these studies show that individual short-term behavioral interventions can be effective for the treatment of PTSD across cultural contexts and age ranges. The three currently available trials of KIDNET for children and adolescents show that KIDNET is a safe, efficient and robust treatment that can be implemented in schools [27], communities [25] and outpatient clinics [26]. However, it is questionable whether the results from these trials can be easily transferred to the current situation of young refugees in Germany. The trial by Catani et al. [27] studied survivors of the war and the tsunami within 4 weeks after the 2009 tsunami catastrophe in Sri Lanka and may be considered as an intervention for acute PTSD rather than chronic war trauma. The study by Ertl et al. [25] included older adolescents as well as young adults and was restricted to child soldiers. The trial of Ruf et al. [26] is probably the most relevant study for the current refugee situation, since it investigated refugee children who had fled to Germany. Even though this study is generally methodologically sound, it is characterized by a small sample size (*N* = 13 per group) and a limited range of outcome measures.

Although current evidence suggests that trauma-focused psychotherapy is probably effective for the treatment of PTSD in war-affected refugee children and adolescents, the provision of effective psychotherapy for traumatized refugee children is currently not a priority within the German health care system and there have been only very limited improvements after the sudden increased influx of refugees in 2015 [31]. Against this background, this trial is urgently needed as it may advance the evidence of the possibilities and limitations of psychotherapy for PTSD and comorbid disorders in refugee children and adolescents. In particular, this trial is characterized by advanced and state-of-the art methods to reduce bias (multi-center trial, blinding of assessors, manualized and standardized treatment, fidelity rating, etc.), an extended range of outcome measures, including depression, physical health, integration parameters, as well as limited exclusion parameters to enhance the external validity of the results.

### Aim of the trial

The YOURTREAT trial is part of the study consortium “Stress, Health and Integration of Young Refugees: Discovering the Interrelations and Improving Access to Healthcare (YOURHEALTH)” funded by the German Federal Ministry of Education and Research (Bundesministerium für Bildung und Forschung; BMBF). This funding agency had no influence on the trial design and will not influence data collection, data management, data analyses, interpretation of study results, publication nor report writing. Within this consortium, the clinical trial YOURTREAT aims to test whether the treatment of young refugees (10–18 years) with KIDNET causes an effective reduction of PTSD and comorbid symptoms. In more detail, KIDNET will be implemented within an innovative complementary health care system that is attached to outpatient clinics of universities and university hospitals. The treatment in the outpatient clinics will be supported by Intercultural Therapy Assistants (ITAs) who will interpret the diagnostic interviews and therapy sessions if required, but can also further support the therapeutic process by accompanying young refugees on their way to and from the therapy, or support the recruitment and eligibility screening into the study. The treatment with 11 sessions of KIDNET supported by ITAs will be compared to treatment as usual (TAU) for young refugees within the general health care system in Germany.

The primary hypothesis of this trial is that, 6 and 12 months after randomization, children who received KIDNET compared to children referred to the regular health care system in the TAU condition will show a greater PTSD symptom reduction. The secondary hypothesis refers to a superiority of KIDNET for the reduction of comorbid depressive, internalizing and externalizing symptoms, as well as suicidal ideation and a reduction of strain caused by discrimination, as well as an improvement of physical health. Furthermore, a lower rate of individuals who still meet the diagnostic criteria of PTSD and a greater response rate is expected in the KIDNET group as opposed to the TAU group.

## Methods

### Trial design

YOURTREAT is a randomized controlled, rater-blinded, two-arm, multi-center, superiority trial with a 1:1 allocation ratio to two parallel groups. Individuals who are screened eligible for the trial will participate in a comprehensive diagnostic interview (t_1_). Among other information (detailed below), this structured interview will assess the diagnosis of PTSD according to the *Diagnostic and Statistical Manual of Mental Disorders, 5th edition* (*DSM-5*). We expect that we will have to interview approximately *N* = 220 children and adolescents (*N* = 55 per site) in order to identify 80 participants (*N* = 20 per site) who fulfill the diagnostic criteria of PTSD. These PTSD patients will be immediately randomized to one of the two arms of the trial; KIDNET or TAU. For individuals included in the KIDNET group, the treatment starts within 1 month after the diagnostic interview and should be terminated at the latest 5 months after randomization. For the beginning and ending of the NET, a tolerance window of 1 week is considered acceptable. Deviations from this time window will be reported. Individuals assigned to TAU will be informed about treatment and counseling options within the local general health care system immediately after the diagnostic interview.

**Fig. 1:**
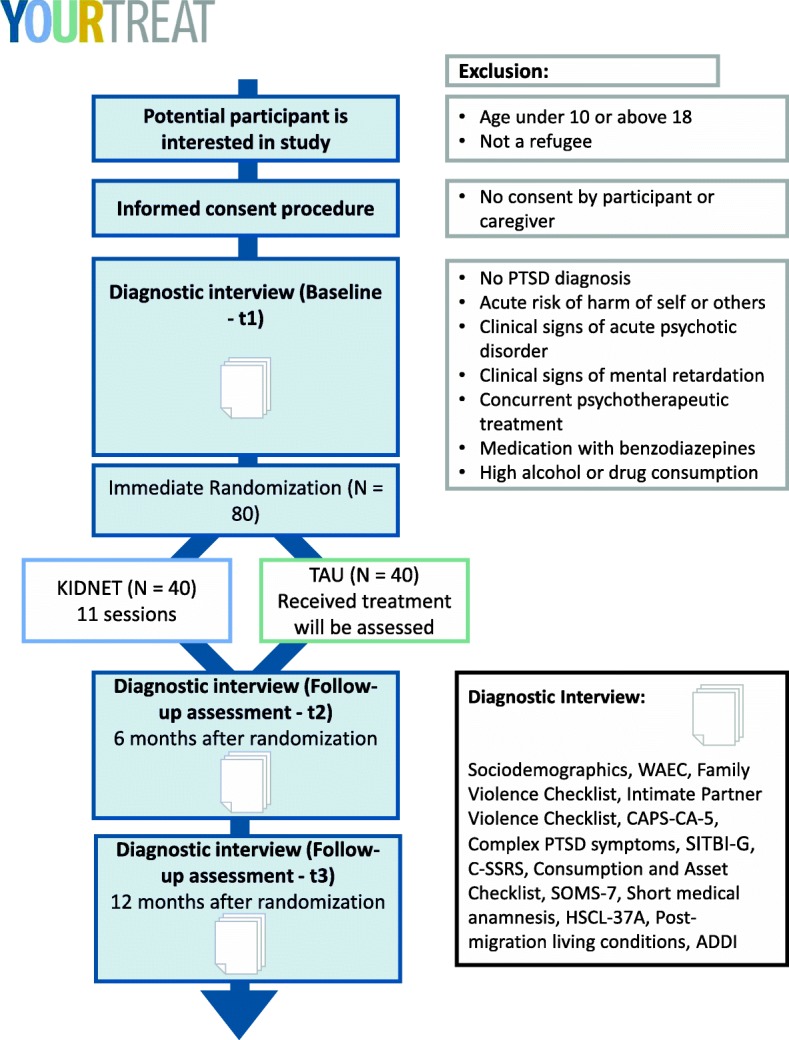
Participant flow of the trial YOURTREAT. Abbreviations: *ADDI* Adolescent Discrimination Distress Index, *CAPS-CA-5* Clinician-Administered PTSD Scale for DSM-5 – Child/Adolescent Version, *C-SSRS* Columbia Suicide Severity Rating Scale, *HSCL-37a* Hopkins Symptom Checklist-37 for Refugee Adolescents, *KIDNET* Narrative Exposure Therapy for Children, *PC-PTSD-5* Primary Care Post-traumatic Stress Disorder Screen for DSM-5, *RHS-15* Refugee Health Screener-15, *SITBI-G,* German version of the Self-Injurious Thoughts and Behaviors Interview, *SOMS-7* Screening for Somatoform Symptoms 7, *TAU* Treatment As Usual, *WAEC* War and Adversities Exposure Checklist

Six months after the diagnostic interview and the randomized group allocation, the first follow-up interview will take place (t_2_). This time point was selected in order to assure a minimum time interval of 1 month between the end of KIDNET and the first follow-up interview. In this way, the diagnostic interview which measures PTSD symptoms in the past month, will not be biased by the experience of symptoms during exposure therapy. The second follow-up interview (t_3_) is scheduled for 12 months after randomization. For the appointments of the follow-up interviews, a tolerance window of ±  2 weeks is considered acceptable. Deviations from this time window will be reported. See Fig. 1 for a schematic overview of the participant flow through the trial. The study protocol was written following the Standard Protocol Items: Recommendations for Interventional Trials (SPIRIT) Statement 2013 [32]. See Additional file 1 for the SPIRIT Checklist. The trial was registered at the German Clinical Trials Registry (Deutsches Register Klinischer Studien; DRKS) on 15 May 2019 (ID: DRKS00017222).

### Outcomes of the trial

#### Primary outcome

The primary outcome of the trial will be the change in PTSD symptom severity from the diagnostic interview before treatment initiation (t_1_) to the follow-up assignments (t_2_ and t_3_). PTSD symptoms will be measured at each assessment by a structured clinical interview using the Clinician-administered PTSD Scale for DSM-5 – Child/Adolescent Version (CAPS-CA-5) [33]. The CAPS-CA-5 represents the “gold standard” for the assessment of PTSD and will be administered by trained psychologists with the help of ITAs who can interpret, if required. In order to standardize the translation of the ITAs, we will translate the CAPS-CA-5 into the most common languages spoken by the patients, e.g., Arabic, Kurdish, Dari and Farsi. Treatment response will be defined as the results of a mixed linear model with the CAPS-CA-5 total symptom score as outcome variable and time and intervention group as well as their interaction as fixed factors. Furthermore, group means (KIDNET vs. TAU) will be compared and the between-group effect sizes (Cohen’s *d)* will be calculated at each follow-up assessment.

#### Secondary outcomes

In addition, the following continuous secondary outcomes will be assessed before treatment (t_1_) and at each follow-up assessment (t_2_ and t_3_). The change of these secondary outcomes will be also evaluated by mixed linear models (see the “Statistics” section for more details).

- Depressive symptoms as well as internalizing and externalizing behavior will be assessed by the Hopkins Symptom Checklist-37 for refugee adolescents (HSCL-37; [34]) The HSCL-37A has been widely used in refugee populations and has shown excellent internal consistency (Cronbach’s alpha = .90) as well as adequate convergent validity with the Strength and Difficulties Questionnaire (SDQ) [34] of *r =* .65 [35]
- Suicidal ideation will be assessed by the Columbia Suicide Severity Rating Scale (C-SSRS) which has a high internal consistency (*α* = .93–.94) and allows for a detailed risk assessment [36]
- Stress caused by discrimination will be assessed by the Adolescent Discrimination Distress Index (ADDI) which exhibited adequate internal consistency for the subscales regarding institutional, educational and peer discrimination (*α* = .60–.72) [37]
- Physical health will be assessed by 17 items selected from the Screening for Somatoform Symptoms 7 (SOMS-7) based on their frequency and sensitivity to change [38]

Finally, the rate of participants who still meet the diagnostic criteria of PTSD according to the CAPS-CA-5, as well as the PTSD response rate will be assessed at each follow-up assessment (t_2_ and t_3_). The PTSD response rate will be defined as the rate of participants who show clinical significant symptom improvement on the CAPS-CA-5, according to the Reliable Change Index (RCI). In the absence of an established value of clinical significance for the DSM-5 and for populations of children and youth, the RCI will be calculated from the study data following the suggestion of Jacobson and Truax [39]. These dichotomous variables will be analyzed by Fisher’s exact tests.

Originally, we also aimed at assessing the secondary outcome acculturation (by the Frankfurt Acculturation Scale for Youth (FRACC-Y) [40]) as an additional parameter of integration. However, during the first diagnostic interviews for the trial, it became evident that the questions were extremely difficult to answer for participants with a high symptom burden and significantly prolonged the duration of the interview. We therefore decided to omit this secondary outcome in order to enhance the quality and validity of the entire interview.

#### Additional measures

Demographic information (including age, gender, information regarding the flight to Germany, religion, education and the current living situation) will be assessed. Furthermore, a short medical anamnesis including addiction will take place at each diagnostic interview.

Adverse and potentially traumatic experiences including family and intimate partner violence as well as war exposure will be assessed by checklists. In more detail, an adapted version of the War and Adversity Exposure Checklist (WAEC) [41] will be employed to assess war-related and general traumatic life events, the short version of the Family Violence Checklist [42] will be used to measure violent experiences within the family, while an unpublished checklist created by our work group will be employed to measure intimate partner violence. Post-migration living conditions for young refugees will be assessed by a brief questionnaire developed by the Neuner Work Group. Furthermore, for accompanied young refugees whose parents agree to participate, parental psychopathology will be assessed by the Refugee Health Screener-15 (RHS-15) [43] and the *Primary Care PTSD Screen for DSM-5* (PC-PTSD-5) [44] and parental behavior by the Parent Behavior Inventory [45]. The RHS has been repeatedly used in refugee populations and shows a high internal consistency (*α* = .95), and good convergent validity (*r* = .88–.91) with diagnostic proxy instruments for anxiety, depression and PTSD [43]. The PC-PTSD-5 offers a more detailed risk assessment for the diagnosis of PTSD and exhibits a high consistency with a clinical diagnosis of PTSD according to the *DSM-5* criteria (diagnostic accuracy of .86 with a cut-off of 3 [44]).

These variables are included in the diagnostic interview as they might impact children’s mental health and treatment success.

#### Exploratory measures

In order to generate hypotheses for forthcoming studies, the following exploratory outcomes will be assessed:

- Economic status and economic behavior, assessed by an adaption of the consumption and asset checklist from Haushofer [46], which was previously employed in low-income countries
- Non-suicidal self-injury, measured by four items of the German version of the self-injurious thoughts and behaviors interview (SITBI-G) [47]
- Diagnosis of complex PTSD, assessed by 13 items following the definition of the proposed *International Classification of Diseases, edition 11* (*ICD-11*) criteria [48]

### Participating centers

YOURTREAT is a multi-center study with four participating centers. Frank Neuner (Bielefeld University) is the principal investigator (PI) of the trial. Treatments will take place at the Refugee Outpatient Clinic for Children and Adolescents at the Medical Center Hamburg Eppendorf (supervised by Areej Zindler), the Outpatient Clinic for Psychotherapy of the University of Konstanz (supervised by Michael Odenwald and Anselm Crombach), the Outpatient Clinic of the Catholic University Eichstätt-Ingolstadt (supervised by Rita Rosner) and the Outpatient Clinic of Bielefeld University (supervised by Frank Neuner). The four study centers have extensive experience with the treatment of children and adolescents, including young refugees. Furthermore, all study centers have established recruitment procedures and referral pathways which can be employed for the purpose of the current trial. Only two of the centers (Bielefeld and Konstanz) had experience with KIDNET before the trial, Eichstätt-Ingolstatt had tried tf-CBT with refugee children before, and Hamburg has applied various approaches to treat young refugees.

### Study sample

#### Subject inclusion and exclusion criteria

Young refugees who fulfill the diagnosis of PTSD according to the *DSM-5* may take part in the study. In more detail, the inclusion criteria are:

- Being a refugee allocated to a community close to one of the participating centers but not necessarily with a permanent permit to stay
- PTSD diagnosis according to CAPS-CA-5
- Age 10–18 years
- Informed consent obtained from participant (if participant is aged ≥ 16 years) or participant and caregiver/legal guardian (if participant is aged < 16 years)

For pragmatic reasons (availability of a sufficient number of ITAs in each language), YOURTREAT will mainly recruit young refugees from Syria, Iraq and Afghanistan, the countries, where the majority of refugees who were seeking asylum in Germany in 2015 originate from.

The following exclusion criteria will be implemented in the trial:

- Acute risk of harm of self or others requiring inpatient treatment
- Clinical signs of acute psychotic disorder
- Clinical signs of mental retardation which would prevent effective psychotherapy
- Concurrent psychotherapeutic treatment
- Anxiolytic treatment with benzodiazepines as this medication is known to interfere with exposure-based psychotherapy (other medication is allowed and will be monitored)
- High alcohol or drug consumption (≥ 2–3 days of consumption per week) represents an exclusion criterion if participants cannot assure their preparedness and confidence to control their consumption in favor of the treatment that they would receive in the framework of the trial

## Procedures

### Recruitment and eligibility screening

All participating centers have extensive experience with the recruitment and treatment of children and adolescents, including young refugees. Participants will be recruited using established recruitment pathways of the specialized outpatient clinics of the centers.

In addition to these conventional recruitment procedures, the trial aims to introduce an innovative entryway for young refugees to psychotherapeutic treatment. In more detail, we plan to train ITAs who cover the most common languages among the current refugee communities, from the refugee and migrant community in each of the four participating. The tasks of the ITAs will be to interpret the diagnostic interviews and therapy sessions for all participants who do not speak German fluently in all study centers. Furthermore, the ITAs can additionally support the therapeutic process as well as the access to psychotherapy by accomplishing the following tasks:

- Accompanying young refugees to and from the therapeutic sessions
- Reducing prejudices against mental health problems and psychotherapy by awareness-raising in the refugee community and de-stigmatization of mental health conditions
- Supporting the recruitment procedure by approaching young refugees (e.g., in schools), by providing basic information about health and mental health, by offering a screening for mental health symptoms, including PTSD, using the Refugee Health Screener (RHS-15) [43] and the *Primary Care PTSD Screen for DSM-5* (PC-PTSD-5) [44], and by making qualified referrals of PTSD cases to the trial or to other specialists in the health care system

The involvement of ITAs in recruitment and psychotherapy will be documented in detail throughout the trial.

### Diagnostic procedures

Diagnostic interviews will be conducted by clinicians with at least a bachelor’s degree with the help of ITAs who interpret the interview. The first diagnostic interview (t_1_) will comprise the assessment of demographic information, a short medical anamnesis including addiction, traumatic and adverse event exposure by means of checklists for war and adversity exposure as well as family and intimate partner violence, and post-migration living conditions. Furthermore, the diagnosis and symptom severity of PTSD will be determined by administering the CAPS-CA-5 [33]. Since our population of young refugees has experienced multiple traumatic experiences, the CAPS-CA-5 symptoms can be answered in relation to several traumatic stressors (and not only to one index trauma). The CAPS-CA-5 will be amended by additional items assessing complex PTSD in the same response format. In order to be eligible for the trial, subjects will have to meet the diagnostic criteria of PTSD according to the *DSM-5* [6]. This structured interview will be succeeded by questionnaires assessing depressive symptoms and internalizing and externalizing symptoms (HSCL-37A) [35], suicidality (C-SSRS) [36], non-suicidal self-injury (four items from SITBI-G) [47], economic status and behavior (adapted from [46]), discrimination (ADDI) [37] and physical health (excerpts from SOMS-7; Rief and Hiller [38]). The follow-up interviews will employ the same questionnaires as detailed above. All diagnostic data will be recorded directly on Case Report Forms (CRFs) and are considered to be source data.

For young refugees who are accompanied by either one parent, both parents, or a legal guardian (who is not an institutional guardian), we aim at assessing mental health symptoms of guardians using the RHS and the PC-PTSD-5. If parents/guardians give their informed consent to participate, they will be screened for mental health symptoms by means of the RHS during each diagnostic interview of the child. Additionally, demographic data of the parents/guardians will also be assessed. Furthermore, we will assess the Parent Behavior Inventory [45] in order to investigate interrelations between parent/guardian mental health, child mental health and parental behavior.

**Fig. 2:**
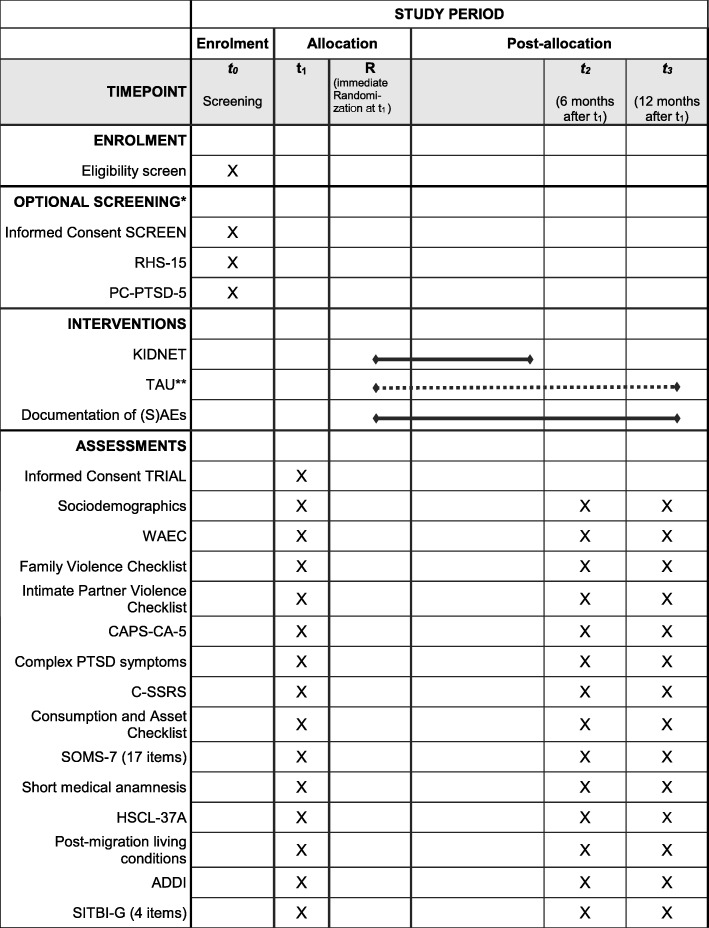
Displayed are the Standard Protocol Items: Recommendations for Interventional Trials (SPIRIT) for the clinical trial YOURTREAT including enrollment, diagnostic assessments and interventions. Abbreviations: *ADDI* Adolescent Discrimination Distress Index, *CAPS-CA-5* Clinician-Administered PTSD Scale for DSM-5 – Child/Adolescent Version, *C-SSRS* Columbia Suicide Severity Rating Scale, *HSCL-37a* Hopkins Symptom Checklist-37 for Refugee Adolescents, *KIDNET* Narrative Exposure Therapy for Children, *PC-PTSD-5* Primary Care Post-traumatic Stress Disorder Screen for DSM-5, *RHS-15* Refugee Health Screener-15, *(S)AE* (Serious) Adverse Event, *SITBI-G* German version of the Self-Injurious Thoughts and Behaviors Interview, *SOMS-7* Screening for Somatoform Symptoms 7, *TAU* Treatment As Usual, *WAEC* War and Adversities Exposure Checklist. *Next to established recruitment procedures of the study centers, participants can be recruited and screened by Intercultural Therapy Assistants as an entryway into the trial. **TAU: patients will be instructed to seek treatment within the general health care system. Therefore, in contrast to KIDNET, the duration of TAU cannot be specified a priori

See the SPIRIT Figure (Fig. 2) for an overview of the procedures of the clinical trial YOURTREAT including enrollment, diagnostic assessments and interventions.

### Interventions

#### Narrative Exposure Therapy for Children (KIDNET)

In the trial, 11 sessions of KIDNET will be provided according to the manual [49]. During the treatment, the child, with the assistance of the therapist, constructs a chronological narrative of their whole life with a focus on the re-construction of the memory of the traumatic events. While the sessions dedicated to the narration of the client’s life are individual sessions with the client, parents or caregivers are invited to participate together with their children in three sessions which concern psychoeducation, current problems and future perspectives.

In the first session (100 min), the parent/caregiver should be present. For the trial, caregivers will be defined as the primary attachment figure. In case of unaccompanied refugee minors, this will be the caregiver who the child trusts the most. If the child or adolescent is unable to identify a single significant caregiver or reports that they does not want a caregiver to participate, this session takes place in individual format with the patient. The first session serves the goal of providing psychoeducation about PTSD and KIDNET, to motivate the caregiver for the continuous support of the treatment, to educate about the internalizing and externalizing stress reactions of the child and about adaptive responses, as well as to provide a continuous monitoring of the safety and well-being of the child throughout the trial. In the second session of KIDNET (100 min), which is conducted with the client alone, the lifeline exercise is performed in order to obtain a first overview of the survivor’s life story. A rope symbolizes the course of life, flowers symbolize positive emotionally arousing events, while stones symbolize negative experiences, including traumatic events. The therapist guides the patient to name the most important life experiences without going into detail. This is succeeded by the development of the narration of the patient’s life from birth to present which may start already in the lifeline session and is continued in the subsequent therapy sessions. While all significant events which were brought up in the lifeline exercise are mentioned in the narration, the most severe traumatic experiences are treated in detail by means of exposure therapy in the following sessions (100 min for each exposure therapy session). However, in contrast to other exposure-based treatments for PTSD which involve repeated exposure to one index trauma, a main focus of KIDNET is the chronological re-construction of autobiographical memory. Furthermore, KIDNET does not contain a variety of therapeutic components, such as relaxation training or cognitive coping, but mainly focuses on the narrative elements. Next to the sessions dedicated to the narration of the client’s life, KIDNET will comprise an additional session of 50 min together with the caregiver that can be scheduled during the course of exposure therapy according to the needs of the patient. It should give the caregiver the opportunity to ask questions regarding the general therapeutic process and to discuss implications of the therapy for daily life. Furthermore, options of how the caregiver can further support the therapeutic process can be discussed. If children or adolescents prefer to discuss everyday problems without the presence of the caregiver, this session will be scheduled with the patient alone. The process of the narration of the client’s life will be finalized by a reflection of the narration of the life story in the 10th session (100 min).

Finally, the last session of the therapy (50 min) will again include the caregiver and serve to celebrate the completion of the treatment as well as to discuss ways of how to cope with residual symptoms and how to further support the client. If desired by the client, the last session can also take place without the caregiver.

#### Treatment as usual (TAU)

Children and adolescents allocated to the TAU group will be referred to the local regular health care system for treatment. Immediately after the communication of group allocation, they will receive detailed information on how to find psychotherapeutic support in their community. For children under the age of 16 years, the psychoeducation on how to find psychotherapeutic support in the community will take place in the presence of the parent or guardian. The interventions provided to the TAU group will be assessed after each follow-up diagnostic assessment.

#### Choice of comparator

The aim of this trial is to test the efficacy of KIDNET for traumatized refugee children and adolescents in specialized outpatient clinics with the help of trained ITAs. For this purpose, it is necessary to compare KIDNET as provided in the specialized outpatient clinics with the efficacy of the treatment that would be available without this module, i.e., a TAU condition. So far, no data exists regarding the efficacy of TAU in this understudied population. Policy-makers often point at the limited evidence regarding the efficacy of trauma treatment for refugees in industrialized countries to justify the limited efforts to invest in the improvement of mental health care for refugees. By comparing a short-term, trauma-focused intervention with TAU for young refugees in Germany, the results of this trial could, therefore, inform major public-health related decisions in Germany; for example, regarding the usefulness of complementary treatment structures for refugees and the necessity of paying costs for interpreters for treatment.

### Measures against bias

#### Randomization

Subjects will be randomly allocated to the two arms of the trial using block-permuted randomization stratified by center based on computer-generated lists that will be provided by the independent statistician. Participants who fulfill the inclusion criteria, including a diagnosis of PTSD, will be immediately assigned to the interventions. Diagnosticians for the baseline diagnostic interview will receive a sequentially numbered, sealed, opaque envelope which will reveal group allocation in case the subject meets the diagnostic criteria for PTSD. The random sequence that determines treatment allocation is kept in a sequence of enumerated sealed envelopes that are provided to the centers. The envelopes will remain sealed to ensure concealment of the sequence of treatment allocations from the research team, assessors and the therapists. At the moment that a patient is assigned to the trial the single corresponding envelope is opened to reveal the allocation of the therapist. Block sizes vary randomly and will not be disclosed in order to assure concealment.

#### Blinding

Due to the behavioral nature of the intervention, neither participants nor therapists and ITAs who interpret the therapy can be blinded. However, the group allocation of the subjects will be concealed from the independent outcome assessors who will conduct the follow-up interviews. At the beginning of the follow-up interviews, participants will be instructed not to reveal any information related to the group that they have been assigned to or regarding the therapeutic process. After the completion of all diagnostic instruments of the follow-up interview, the independent assessors will complete a questionnaire asking whether any information regarding group allocation was revealed in the diagnostic interview. Cases of premature unblinding will be documented and reported.

#### Measures taken to prevent bias in assessment

The diagnostic interviews will be conducted by specially trained assessors. As the CAPS-CA-5 [33] serves as the primary outcome of the study, assessors are extensively trained in this instrument. During the trial, all interviews will be videotaped and a random selection of 10% per site will be checked and rated by an independent evaluator to assess interrater reliability of the CAPS-CA-5.

#### Treatment fidelity

Treatments with KIDNET will be conducted by expert clinicians (minimum bachelor’s degree) specialized in the field of psychotraumatology. All study therapists receive a training in KIDNET which includes a 2-day basic training including the theoretical background of the treatment, the practical implementation of KIDNET, case demonstrations, practical exercise and role-plays in small groups under the supervision of experienced KIDNET therapists. This initial training is followed by an advanced training which includes the discussion and review of test cases. Furthermore, treatment fidelity during the trial will be assured by external NET case consultation with experienced NET therapists after at least every fourth therapy session as well as regular supervision and intervision meetings at the study centers. The frequency of supervision and intervision meetings at the sites will be recorded throughout the study. In addition, all sessions will be videotaped and a random selection of 10% per site will be checked by independent raters for consistency with the treatment manual. Finally, 10% of the therapy session sheets (which assess the date, duration, main topics covered in the session, as well as difficulties in the KIDNET process) of all centers will be reviewed. Observed inconsistencies with the treatment manual will be documented, but do not constitute an exclusion criterion for the trial.

#### Measures taken to avoid attrition bias

In order to avoid systematic drop-out from the study, participants will receive compensation of 20€ in the form of a voucher for each follow-up interview. Furthermore, the inclusion of the caregivers and ITAs in the therapeutic process is intended to increase the cultural acceptance of the intervention as well as the reliability of the children and adolescents to show up for the sessions and diagnostic interviews. Finally, we will analyze all trial participants as randomized (intention-to-treat analyses).

### Data management

Data will be collected at the study centers according to the General Data Protection Regulation (GDPR). The PI will appoint and supervise a Data Management Team (DMT) responsible for all aspects related to data storage, data integrity and data monitoring during the project period. Data storage and transfer will be conducted exclusively in an encrypted manner.

The centers will transfer the pseudonymized encrypted data to a secured server accessible by the DMT. The DMT will create a merged, pseudonymized dataset only accessible by themselves which will be stored for the project duration. Checks for completeness as well as range checks will be conducted to ensure data integrity. Furthermore, the DMT will perform at least one site-monitoring per year at each participating center where access will be granted to source documents in order to check for completion of informed consent sheets and consistency with the entered data.

Statistical analyses will be conducted by the independent certified biostatistician Theodor May, who will also supervise the DMT in accordance with the GDPR. For long-term storage, the anonymized dataset (after deletion of the code list and database lock) will be stored in the Bielefeld University data repository and provided to external scientists upon request for further analysis.

## Statistics

### Power analysis and sample size calculation

The Ruf et al. pilot study [26] achieved a treatment effect size of 1.9 for NET and 0.3 for the waiting-list control group. However, we expect that the contrast will be smaller for this study, as we will have an active control condition and will provide detailed information to children and adolescents from the TAU condition on how to seek treatment in the regular health care system. Therefore, we base the effect size calculation at a conservative estimation of a between-group effect size of *d* = 0.6. To test the hypothesis that a treatment with KIDNET is more effective than TAU, with a one-sided alpha level set at 0.05 and a power level of 0.8, we need to enroll *N* = 72 subjects (*N* = 36 per group) into the present study. Once randomized, loss to follow-up was small (generally lower than 5%) in the Ruf et al. study [26] as well as in other NET studies [50]. Most importantly, as the main analysis of the primary outcome measure will be calculated using a mixed-effects model, all randomized cases can be included in the statistical analyses. Therefore, the intended sample size of *N* = 80 will be sound to answer the research questions of the trial.

### Data analyses

The confirmatory analysis of this study will be calculated as a mixed-effects model with the CAPS-CA-5 score as the outcome variable. Mixed models are especially suited for longitudinal studies as they can account for serial correlation within participants, are relatively robust to randomly missing data, and can incorporate certain nonrandom missing data without biasing model estimates. In detail, participants will be modeled as a random factor (including random intercepts or random intercepts and slopes), while time and intervention (KIDNET vs. TAU), as well their interaction, will be modeled as fixed factors. The hypothesis that KIDNET is superior to TAU in the treatment of PTSD will be evaluated by the significance test of the interaction effect time × intervention.

In case of a significant interaction effect, two planned general linear hypotheses will be calculated as post-hoc tests for linear mixed-effect models in order to test between-group differences at t_2_ and t_3_. One-sided *p* values will be adjusted for multiple comparisons following the Holm procedure.

We will perform an intention-to-treat analysis; that is, all trial participants will be analyzed as randomized, even if they discontinue treatment or are unavailable for one or both of the follow-up interviews. The between-group effect size (Cohen’s *d*) will be calculated at each follow-up assessment (t_2_ and t_3_). The intention-to-treat analysis will be supplemented by a modified intention-to-treat analysis which will include only those participants who participated in at least one post-randomization diagnostic interview. Furthermore, participants who were randomized to KIDNET will be included only if they participated in at least one KIDNET therapy session.

Continuous secondary outcome measures are analyzed in the same way. In the absence of a valid cut-off score for clinically significant change or treatment response of the CAPS-CA-5, the rate of subjects with clinically significant improvement as well as worsening based on the RCI will be compared between groups using Fisher’s exact tests at the follow-up assessments t_2_ and t_3_, separately. For this purpose, the RCI will be calculated based on the pre-treatment scores of the study sample following the suggestion of Jacobson and Truax [39]. Missing values due to premature withdraw will be considered as treatment failure (classified as no response).

In addition, in case of significant treatment effects, center effects regarding the primary efficacy endpoint are investigated. For this purpose, the change in CAPS-CA-5 at t_3_ (compared to baseline t_1_) are analyzed exploratively using a mixed-effects model that includes participants as a random factor and center, intervention and center × intervention as fixed factors. The significance level will be set at 0.05 for all analyses.

### Safety and ethical aspects of the trial

The trial procedures follow the Declaration of Helsinki and the ICH Guidelines for Good Clinical Practice (ICH-GCP) [51]. The Ethics Committee of the German Psychological Association (Deutsche Gesellschaft für Psychologie, DGPs) approved the study procedures (approval date: 6 May 2019). Modifications of the protocol, which are not merely of administrative nature, such as minor corrections or clarifications, and which might impact patient safety, ethical aspects of the trial or the conduct, and scientific evaluation of the trial, will be submitted as protocol amendments to the Ethics Committee and require approval. Furthermore, the record at the trial registry DRKS will be updated and the BMBF will be informed in case of protocol amendments. Such amendments will be agreed upon by the PI after consultation with the External Advisory Board.

### Safety aspects

So far, no trial has ever reported serious adverse events (SAEs) caused by psychotherapy of trauma-related disorders in children and adolescents [cf. 52, 53]. Since the planned treatment study can be, therefore, considered as safe, we refrain from installing an external Safety Monitoring Committee for pragmatic reasons. Instead, we have appointed a SAE Management Committee constituted of the lead investigators at the sites (Areej Zindler, Rita Rosner, Anselm Crombach, Michael Odenwald), the PI (Frank Neuner), the independent biostatistician and the External Advisory Board of the trial who will supervise the safety of the study.

The following dangerous occurrences will be defined as SAEs in accordance to the ICH-GCP and will be closely monitored during treatment, and at the follow-up assessments:

- Attempted suicide
- Event which results in death (suicide or other)
- Life-threatening event
- Event which results in significant disability

All such dangerous events and other safety aspects will be forwarded to the SAE Management Committee. In addition, the independent biostatistician will conduct interim analyses after 30% and 50% of the completed 6-month follow-ups in order to monitor the symptom development as well as the occurrence of potential SAEs. If an SAE is identified, the SAE Management Committee will investigate whether there is a causal link between the trial and the SAE. If the SAE Management Committee decides that further trial participation would be a safety concern for the patient, the patient will be withdrawn from the study. In the case of serious safety concerns, the trial will be stopped.

### Informed consent procedure

Trained psychologists or physicians (minimum bachelor’s degree) will introduce the study procedures to the participants. Our study population of young refugees is particularly vulnerable, as their age as well as their limited knowledge of the German health system might impair their ability to understand the aims and procedures of the study. In order to assure the comprehension of the information of the informed consent, and to standardize the informed consent between the centers, we have decided to supplement the written informed consent for the trial by a video which reads out the exact wording of the informed consent. Furthermore, the informed consent is written in easy language. The written informed consent will be available in the languages of the participants. They will detail the study procedures including diagnostic interviews, randomization, treatment options, potential temporary distress after interviews or therapeutic sessions, the possibility of terminating study participation without any disadvantage, and data management. Trained psychologists or physicians (minimum bachelor’s degree) will answer questions arising, and will subsequently obtain written informed consent from subjects who decide to participate in the trial. In addition, participants will be asked whether they want to give informed consent to be contacted for the participation in ancillary studies. They will be informed that this decision does not affect their participation in the YOURTREAT trial.

### Coping with distress

Similar to the re-experiencing symptoms that PTSD patients experience in everyday life, patients might also experience high and distressing levels of anxiety or other unpleasant emotional reactions during assessments or sessions of exposure-based psychotherapy. However, in contrast to their everyday experience, where they usually have to deal with these symptoms on their own, the assessor/therapist can help them to cope with these reactions, e.g., by reinforcing reality or by providing psychoeducation.

### Post-trial care

Individuals assigned to the TAU condition who still suffer from PTSD will be offered a treatment with KIDNET or an equally effective therapy within the participating outpatient clinics. Furthermore, participants who do not benefit from KIDNET (i.e., still suffer from PTSD at the last follow-up) will be offered additional treatments at the participating centers or will be referred to appropriate inpatient or outpatient treatments.

## Discussion

In spite of the known detrimental consequences of trauma originating from war and flight on the mental and physical health of refugees and their integration into the host culture, the evidence regarding effective treatment options for this vulnerable population is very limited [54]. As presented in the literature review in the introduction, research regarding effective interventions for *young* refugees is even sparser. Furthermore, different barriers including language difficulties, fear of stigma, and limited knowledge about treatment options for mental health disorders make it difficult for refugees to seek for help. This is aggravated by bureaucratic obstacles for psychotherapists to receive payments for psychotherapy delivered to refugees with unsecure asylum status, and the required interpreters. Finally, many practitioners are hesitant to deliver evidence-based PTSD treatments due to fears of symptom aggravation or treatment discontinuation [55, 56]. In order to counteract these barriers, and to improve the mental health care for young refugees in the German health care system, systematic studies are urgently required to inform the scientific and clinical community as well as public health decisions.

The lack of systematic studies on effective interventions for war-affected refugee children and adolescents suffering from PTSD might have several reasons. First of all, the group of adolescents might be of greater risk of dropping out of treatment trials due to the developmental tasks in this age period, and unstable mood and motivation [57]. Even more, young refugees face unstable living and housing situations and might be relocated at short notice, making it even more difficult to prevent attrition from the trial. Furthermore, a trial with young refugees requires a close collaboration with, and intense training of, interpreters from different cultures, demanding additional resources from the research team.

Against this background, the current trial aims at providing the required knowledge regarding effective treatment options for young refugees. Strengths and innovative aspects of the trial include (1) the unique study sample of young refugees hosted in Germany, (2) the systematic assessment of PTSD with the CAPS-CA-5, the gold standard in the diagnosis of PTSD, (3) the wide range of outcome measurements, (4) the limited exclusion criteria, which enhance the external validity of the trial, (5) the short and pragmatic nature of the delivered treatment, (6) an evaluation of the usual treatment (TAU) within the German health care system and (7) the implementation and evaluation of a support structure by ITAs, who can interpret and support the therapeutic process.

A potential limitation of the trial could be the danger of drop-outs due to the instability of the investigated population. However, a previous study with young refugees treated with NET also managed to achieve a low attrition rate [26]. Furthermore, we intend to increase the retention of subjects by closely collaborating with ITAs from the community of the study participants as well as with the caregivers of the young refugees.

In addition, we have to note that the trial is led by Frank Neuner, one of the developers of (KID)NET. Therefore, a high treatment loyalty could be stated as a limitation of the trial. However, it is important to note that two of the three other centers taking part in the study have no prior experience with (KID)NET and previously predominantly used other treatment approaches for trauma-related disorders. This increases the external validity and generalizability of the trial.

In sum, this trial has the potential for a scientific impact on major public-health-related decisions in Germany; for example, regarding the usefulness of complementary treatment structures for refugees and the necessity to pay costs for interpreters for treatment. In addition, we expect that this trial can inform the regular health care system (clinics, psychotherapists in outpatient clinics, psychotherapist in private practices) as well as the treatment centers for refugees about the effect of short-term therapy for refugee children and adolescents and, ultimately, improve the quality of care. As there is a lack of methodological sound RCTs for refugee children, we expect that this trial can have a major impact on the guidelines for the treatment of PTSD.

### Dissemination

The trial results will be disseminated to the scientific community by publications in international peer-reviewed journals. The results regarding the primary outcome of the trial will be published regardless of the direction and statistical significance of the effect. Next to the publication of the results in scientific journals, we will communicate the results to national and international psychological and psychiatric associations as well as to the policy-makers in Germany, and the public media.

### Trial status

Protocol version 1.5, 19 October 2019.

Recruitment into the trial started in June 2019.

Recruitment will be completed in approximately May 2021.

## Supplementary information

**Additional file 1.** Standard Protocol Items: Recommendations for Interventional Trials (SPIRIT) 2013 Checklist: recommended items to address in a clinical trial protocol and related documents*

## Data Availability

Not applicable

## Abbreviations

ADDI: Adolescent Discrimination Distress Index
AE: Adverse Event
CAPS-CA-5: Clinician-Administered PTSD Scale for DSM-5 – Child/Adolescent Version
CBT: Cognitive Behavior Therapy
CRF: Case Report Form
C-SSRS: Columbia Suicide Severity Rating Scale
DMT: Data Management Team
DSM-5: Diagnostic and Statistical Manual of Mental Disorders, 5th edition
GCP: Good Clinical Practice
GDPR: General Data Protection Regulation
HSCL-37A: Hopkins Symptom Checklist-37 for Refugee Adolescents
ICH: International Council for Harmonization
ITA: Intercultural Therapy Assistant
ITT: Intention-to-treat
KIDNET: Narrative Exposure Therapy for Children
NET: Narrative Exposure Therapy
PC-PTSD-5: Primary Care PTSD Screen for DSM-5
PI: Principal Investigator
PTSD: Post-Traumatic Stress Disorder
RCI: Reliable Change Index
RHS-15: Refugee Heath Screener-15
SAE: Serious Adverse Event
SITBI-G: German version of the Self-Injurious Thoughts and Behaviors interview
SOMS-7: Screening for Somatoform Symptoms 7
SPIRIT: Standard Protocol Items: Recommendations for Interventional Trials
TAU: Treatment As Usual
WAEC: War and Adversity Exposure Checklist
